# Knockout of adenylosuccinate synthase *purA* increases susceptibility to colistin in *Escherichia coli*

**DOI:** 10.1093/femsle/fnae007

**Published:** 2024-02-01

**Authors:** Tomonori Kano, Kazuya Ishikawa, Kazuyuki Furuta, Chikara Kaito

**Affiliations:** Graduate School of Medicine, Dentistry, and Pharmaceutical Sciences, Okayama University, 1-1-1 Tsushima-naka, Kita-ku, Okayama 700–8530, Japan; Graduate School of Medicine, Dentistry, and Pharmaceutical Sciences, Okayama University, 1-1-1 Tsushima-naka, Kita-ku, Okayama 700–8530, Japan; Graduate School of Medicine, Dentistry, and Pharmaceutical Sciences, Okayama University, 1-1-1 Tsushima-naka, Kita-ku, Okayama 700–8530, Japan; Graduate School of Medicine, Dentistry, and Pharmaceutical Sciences, Okayama University, 1-1-1 Tsushima-naka, Kita-ku, Okayama 700–8530, Japan

**Keywords:** colistin, adenylosuccinate synthase, *de novo* purine synthesis, membrane potential, ATP synthesis

## Abstract

Colistin is a cationic cyclic antimicrobial peptide used as a last resort against multidrug-resistant gram-negative bacteria. To understand the factors involved in colistin susceptibility, we screened colistin-sensitive mutants from an *E. coli* gene-knockout library (Keio collection). The knockout of *purA*, whose product catalyzes the synthesis of adenylosuccinate from IMP in the *de novo* purine synthesis pathway, resulted in increased sensitivity to colistin. Adenylosuccinate is subsequently converted to AMP, which is phosphorylated to produce ADP, a substrate for ATP synthesis. The amount of ATP was lower in the *purA*-knockout mutant than that in the wild-type strain. ATP synthesis is coupled with proton transfer, and it contributes to the membrane potential. Using the membrane potential probe, 3,3′-diethyloxacarbocyanine iodide [DiOC2(3)], we found that the membrane was hyperpolarized in the *purA*-knockout mutant compared to that in the wild-type strain. Treatment with the proton uncoupler, carbonyl cyanide m-chlorophenyl hydrazone (CCCP), abolished the hyperpolarization and colistin sensitivity in the mutant. The *purA*-knockout mutant exhibited increased sensitivity to aminoglycosides, kanamycin, and gentamicin; their uptake requires a membrane potential. Therefore, the knockout of *purA*, an adenylosuccinate synthase, decreases ATP synthesis concurrently with membrane hyperpolarization, resulting in increased sensitivity to colistin.

## Teaser

Disruption of de novo purine synthesis pathway leads to decreased ATP synthesis and hyperpolarization of the bacterial cell membrane, resulting in increased susceptibility to colistin.

## Introduction

The emergence of antibiotic-resistant bacteria is a serious medical concern. More than one million people died from antibiotic-resistant bacterial infections worldwide in 2019 (Antimicrobial Resistance Collaborators 2022), indicating the urgent need for developing strategies to overcome antibiotic resistance. Colistin is used against multidrug-resistant gram-negative bacteria, such as *Pseudomonas aeruginosa* and *Acinetobacter baumanii*, as a last-resort antibiotic (Kassamali et al. 2015, El-Sayed Ahmed et al. 2020). Colistin has a positive charge, and it electrostatically interacts with LPS, disrupting the outer membrane; in addition, the hydrophobic moiety of colistin disrupts the inner membrane (Velkov et al. 2010, El-Sayed Ahmed et al. 2020). The molecular mechanism underlying colistin action remains unclear. LPS modification by two-component systems, such as PmrAB and PhoPQ, induces bacterial resistance to colistin by inhibiting the interaction between colistin and the outer membrane (El-Sayed Ahmed et al. 2020).

Bacterial membrane potential influences the susceptibility to aminoglycosides and polymyxin B, which are positively charged antimicrobials, because electrostatic interactions between these antimicrobial molecules and the bacterial cell membrane are key factors for their uptake (Taber et al. 1987, Alteri et al. 2011). In *Staphylococcus aureus*, ATP synthase knockout leads to the hyperpolarization of the bacterial membrane, resulting in susceptibility to polymyxin B and colistin (Vestergaard et al. 2017). Thus, gene mutations affecting membrane potential can alter the susceptibility to colistin; however, the genes affecting both colistin susceptibility and membrane potential are not fully understood.

In this study, we screened colistin-sensitive mutants from an *E. coli* gene knockout library to identify genes that influence colistin susceptibility. The knockout of *purA*, which encodes adenylosuccinate synthase that is responsible for the *de novo* adenine synthesis from IMP (Jensen et al. 2008), results in a colistin-sensitive phenotype; *purA* knockout induces colistin sensitivity by altering the membrane potential.

## Materials and methods

### Bacterial strains and culture conditions

*Escherichia coli* BW25113 and its gene-knockout mutant were grown in Luria–Bertani (LB) agar plates. A single colony was inoculated into the LB liquid medium and aerobically cultured at 37°C with agitation. *Escherichia coli* strains transformed with pMW118 were cultured in the LB liquid medium supplemented with 100 µg/mL ampicillin and 1 mM IPTG. The bacterial strains and plasmids used in this study are listed in Table S1.

### Screening of colistin-sensitive strains

*Escherichia coli* gene-knockout mutants from the Keio library (Baba et al. 2006, Yamamoto et al. 2009) were cultured in LB medium supplemented with 50 µg/mL kanamycin in a 96-well microplate, with shaking using a microplate shaker (BR-034P, Taitec), at 37°C overnight. The overnight cultures were inoculated into a fresh LB medium supplemented with 200 ng/mL colistin in a 96-well microplate and were cultured at 37°C for 24 h without shaking. The OD_595_ values of the cultures were measured using a microplate reader (Multiskan FC, Thermo Fisher Scientific).

### Bacterial killing assay

*Escherichia coli* overnight culture was diluted to 1 × 10^6^ CFU/mL in LB medium. For the colistin-killing assay, the diluted culture was supplemented with 0.8 µg/mL colistin and was incubated at 37°C for 30 min without shaking. The culture was serially diluted 10-fold and spread on an LB agar plate and incubated overnight at 37°C. The colonies were counted to calculate the number of surviving bacteria. For the aminoglycoside-killing assay, the diluted culture was supplemented with 17.5 µg/mL kanamycin or 10 µg/mL gentamicin and incubated at 37°C for 60 min without shaking. The culture was serially diluted 10-fold; 5 µL was spotted onto LB agar plates, which were then incubated overnight at 37°C.

### Genetic manipulation

To obtain the *purA*-gene knockout mutant, transduction with P1 *vir* was performed using JW4135-KC as the phage donor and BW25113 as the recipient strain. The kanamycin-resistant marker in the transductant was deleted by introducing pCP20 expressing FLP recombinase, resulting in the *purA*-gene knockout mutant (markerless deletion). To construct a complementation plasmid, a DNA fragment carrying the *purA* gene was amplified via PCR using the genomic DNA of BW25113 as the template and specific primers (Table S2); the amplified gene was inserted into the KpnI and BamHI sites of pMW118, resulting in pMW118-purA.

### Measurement of the membrane potential

Membrane potentials were measured as described previously (Hudson et al. 2020) with minor modifications. An aliquot (50 µL) of the overnight culture was inoculated into 5 mL fresh LB medium supplemented with 100 µg/mL ampicillin and 1 mM IPTG and was cultured with shaking until an OD_600_ of 0.5 was reached. The cultures (1 mL) were centrifuged to collect bacterial cells; the cells were suspended in PBS. EDTA (10 mM) was added to the cell suspension; the suspension was incubated at room temperature for 5 min and then centrifuged. The cells were suspended in a buffer (500 µL) (130 mM NaCl, 60 mM Na_2_HPO_4_, 60 mM NaH_2_PO_4_, 10 mM glucose, 5 mM KCl, 0.5 mM MgCl_2_); 30 µM DiOC2(3) with or without 1 mM CCCP was added to the cell suspension. The cells were washed with the same buffer, and fluorescence (RFU/s) was measured using a fluorescence microplate reader (Fluoroskan Ascent CF, Thermo Fisher Scientific) with excitation at 485 nm and emission at 678 nm.

### Measurement of cell surface charge by cytochrome C binding assay

Cell surface charge was measured as described previously (Gasch et al. 2013) with minor modifications. Overnight cultures (5 mL) were centrifuged to collect the bacterial cells; they were washed twice with buffer (20 mM MOPS, 5 mM sodium citrate, 1 mM EDTA, pH7.0). The cells were suspended in the same buffer and the OD_600_ was adjusted to 21; 500 µg/mL cytochrome C was added to the cells and incubated at 37°C for 15 min. The cells were centrifuged, and the OD_530_ value of the supernatant was measured. The concentration of cytochrome C in the supernatant was calculated using a standard curve.

### Measurement of zeta potential

Overnight cultures of *E. coli* were diluted 500-fold using PBS. The zeta potential was measured using Zetasizer Nano ZSP (Malvern) and disposable folded capillary cells (DTS1070, Malvern). Equilibration was performed for 300 s before the measurement of each sample.

### Quantifying ATP

ATP levels were measured as described previously (Ryuno et al. 2019) with minor modifications. Overnight cultures (50 µL) were inoculated into fresh LB medium (5 mL) supplemented with 100 µg/mL ampicillin and 1 mM IPTG and cultured to OD_600_ = 0.5 or 0.8. The culture (100 µL) was mixed with an equal volume of ethanol and incubated on ice for 15 min. The sample was centrifuged, and the supernatant was diluted 100-fold with Milli-Q water. The sample (50 µL) was mixed with an equal volume of solution containing firefly luciferase and the substrate (Kikkoman, Japan); the luminescence was measured immediately using a luminometer (Promega).

### Statistical analysis

All data were analyzed using one-way ANOVA with post hoc Dunnett's test using Prism 9 (GraphPad software).

## Results

### *purA* knockout increased susceptibility to colistin

**Figure 1.**
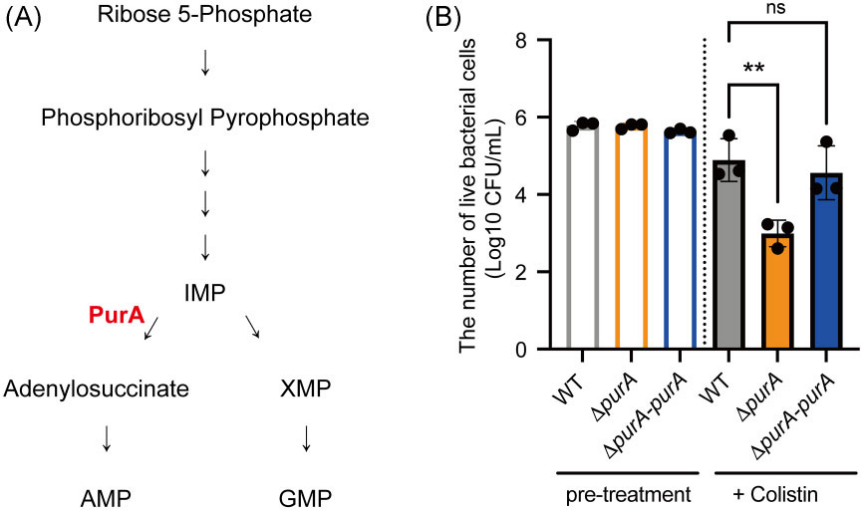
*purA* knockout increases susceptibility to colistin. (A) *De novo* purine synthesis pathway (B) Bacterial cells (1 × 10^6^ CFU/mL) were treated with 0.8 µg/mL colistin for 30 min. The number of viable bacterial cells before and after treatment was measured by plating the samples on LB agar plates and incubating the plates overnight. The data are presented as means ± SD of three independent experiments. **, *P* < 0.01. ns: not significant.

We searched for colistin-sensitive mutants in the *E. coli* gene knockout library (Keio collection) (Baba et al. 2006) and identified 21 gene knockout mutants that could not grow in the presence of 200 ng/mL colistin (Table S3). There were mutants of *atpB, atpE*, and *atpH* genes encoding ATP synthase subunits (Table S3). We focused on PurA, an enzyme in the *de novo* purine synthetic pathway (Fig. 1A) that plays an important role in bacterial virulence (Ivanovics et al. 1968, Sigwart et al. 1989, Faith et al. 2012, Connolly et al. 2017) and analyzed the mechanism of colistin susceptibility. To confirm the increased susceptibility of the *purA-*knockout mutant to colistin, we performed a bactericidal assay. Following treatment with colistin for 30 min, the number of viable bacterial cells was 100-fold lower in the *purA*-knockout mutant compared to that in the wild-type strain (Fig. 1B). The number of viable bacterial cells in the *purA*-knockout mutant was restored by the introduction of *purA* (Fig. 1B). Therefore, *purA* knockout increases colistin susceptibility.

### *purA* knockout decreased the amount of ATP

**Figure 2.**
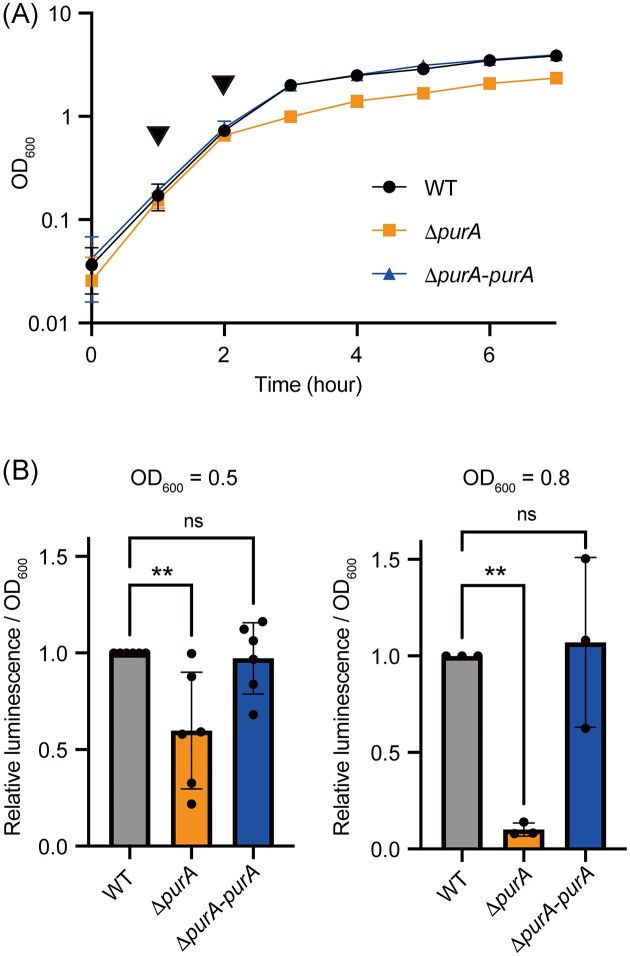
*purA* knockout decreases the amount of ATP. (A) Growth curves of wild-type and *purA-*knockout mutants. Arrowheads indicate the time point (OD_600_ = 0.5 and OD_600_ = 0.8) at which samples were taken for analysis in (B). The data are presented as means ± SD of three independent experiments. (B) The amount of ATP in the wild-type strain and *purA*-knockout mutant was measured. The data are presented as means ± SD of six independent experiments at OD_600_ = 0.5 experiments and three independent experiments at OD_600_ = 0.8 experiments. **, *P* < 0.01. ns: not significant.

We speculated that *purA* knockout reduces AMP levels, resulting in the depletion of ADP and ATP. We examined the growth differences between the wild-type strain and the *purA*-knockout mutant. The growth of *purA*-knockout mutant was comparable to that of the wild-type strain until the late exponential phase (OD_600_ = 0.8); however, the growth was delayed, compared to that of the wild-type strain, from the late exponential phase to the stationary phase (Fig. 2A). We measured the amount of ATP per OD_600_ value at the mid and late exponential phases (OD_600_ = 0.5 and 0.8). The amount of ATP was lower in the *purA*-knockout mutant than that in the wild-type strain, at both growth time points (Fig. 2B). The amount of ATP in the *purA*-knockout mutant was restored by the introduction of *purA* (Fig. 2B). Therefore, *purA* knockout decreases ATP levels in bacterial cells.

### *purA* knockout caused hyperpolarization of the bacterial membrane

**Figure 3.**
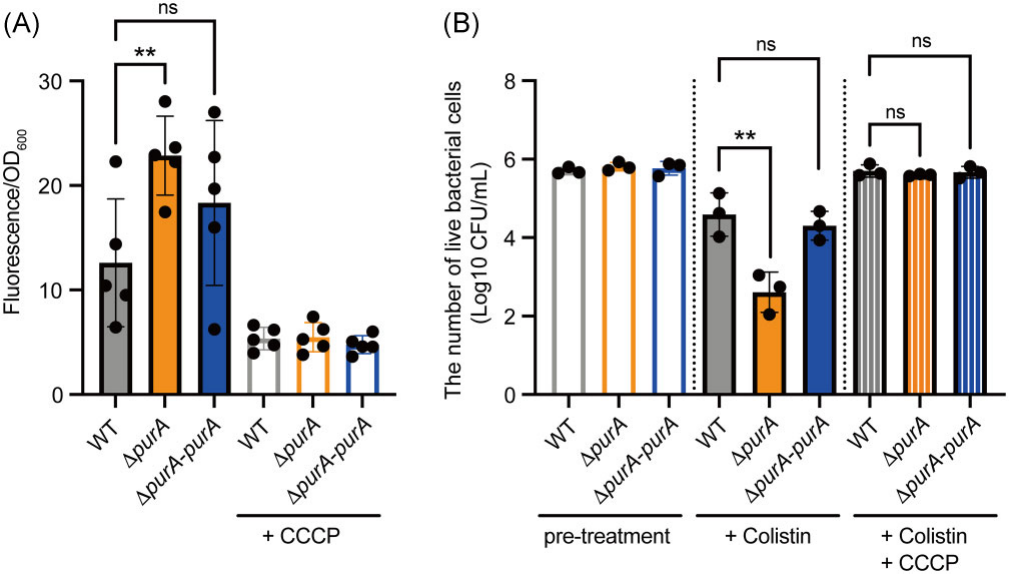
*purA* knockout leads to membrane hyperpolarization. (A) Exponentially growing *E. coli* cells were treated with or without 1 mM CCCP, stained with DiOC2(3), and the fluorescence was measured. The data are presented as means ± SD of five independent experiments. **, *P* < 0.01. ns: not significant. (B) *E. coli* cells (1 × 10^6^ CFU/mL) were treated with 0.8 µg/mL colistin with or without 50 µM CCCP for 30 min. The number of viable bacterial cells was determined. The data are presented as means ± SD of three independent experiments. **, *P* < 0.01. ns: not significant.

ATP synthesis is coupled with proton transfer, which contributes to the membrane potential. We hypothesized that *purA* knockout decreases ATP synthesis concomitantly with decreased proton transfer, leading to bacterial membrane hyperpolarization. We examined the membrane potential using the membrane potential probe, 3,3′-diethyloxacarbocyanine iodide [DiOC2(3)] (Hudson et al. 2020). The *purA*-knockout mutant showed a higher fluorescence intensity than the wild-type strain (Fig. 3A). The increased fluorescence intensity in the *purA*-knockout mutant was abolished by the introduction of *purA* (Fig. 3A). In addition, the fluorescence signal of DiOC2(3) decreased after treatment with the proton uncoupler (CCCP) (Fig. 3A). Therefore, *purA* knockout led to membrane hyperpolarization.

Next, we examined whether treatment with CCCP abolished colistin susceptibility in the *purA*-knockout mutant. The CCCP treatment abolished the difference in colistin susceptibility between the *purA*-knockout mutant and wild-type strains (Fig. 3B). These results suggested that the colistin susceptibility of the *purA*-knockout mutant could be attributed to membrane hyperpolarization.

### *purA* knockout increased susceptibility to aminoglycosides

**Figure 4.**
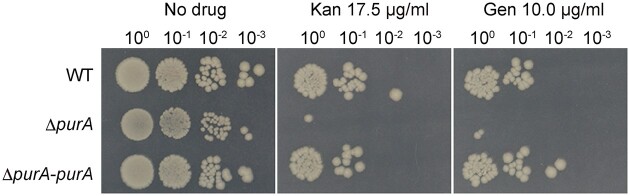
*purA* knockout increases susceptibility to aminoglycosides. *E. coli* cells (1 × 10^6^ CFU/mL) were treated with 17.5 µg/mL kanamycin or 10 µg/mL gentamicin for 1 h. The sample was serially diluted 10-fold, spotted onto LB agar plates, and incubated overnight at 37°C.

Membrane potential influences the bacterial susceptibility to cationic antimicrobial peptides and aminoglycosides (Taber et al. 1987, Alteri et al. 2011, Liu et al. 2020); thus, we examined whether the *purA-*knockout mutant was sensitive to aminoglycosides, using a bactericidal assay. Following treatment with kanamycin or gentamicin, the number of viable bacterial cells was lower in the *purA*-knockout mutant than that in the wild-type strain (Fig. 4). The number of viable bacterial cells in the *purA*-knockout mutant was restored by the introduction of *purA* (Fig. 4). Therefore, *purA* knockout sensitizes *E. coli* to aminoglycosides.

### *purA* knockout did not alter the cell surface charge

**Figure 5.**
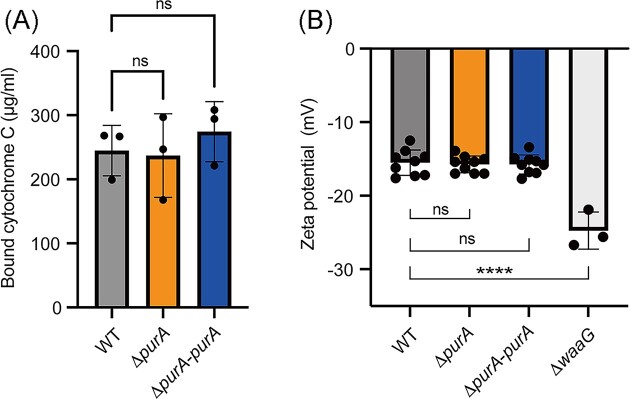
*purA* knockout does not alter cell surface charge. (A) Overnight-cultured *E. coli* cells were mixed with cytochrome C, and the amount of cytochrome C bound to the bacterial cells was measured. The data are presented as means ± SD of three independent experiments. ns: not significant. (B) Overnight-cultured *E. coli* cells were suspended in PBS and the zeta potential was measured. The data are presented as means ± SD of at least three biological replicates. ^****^, *P* < 0.0001. ns: not significant.

Alteration of cell surface charge following LPS modification influences susceptibility to cationic antimicrobial peptides, such as colistin (El-Sayed Ahmed et al. 2020). We examined the cell surface charge in the *purA*-knockout mutant using a binding assay for cytochrome C, a positively charged protein. The binding of cytochrome C did not differ between the wild-type strain and the *purA*-knockout mutant (Fig. 5A). Furthermore, to measure the cell surface charge using another analytical method, we examined the zeta potential of bacterial cells and observed no significant difference in the zeta potential between the wild-type strain and the *purA*-knockout mutant (Fig. 5B). We also tested the *waaG*-knockout mutant as a positive control, we found that the mutant exhibited lower zeta potential than that of the wild-type strain, consistent with previous reports (Hyldgaard et al. 2014, Soh et al. 2020). These results suggested that the colistin sensitivity in the *purA-*knockout is not related to alterations in the cell surface charge.

## Discussion

*purA* knockout increased bacterial susceptibility to colistin. Membrane potential was hyperpolarized in the *purA-*knockout mutant, and CCCP abolished both membrane hyperpolarization and colistin susceptibility in the *purA-*knockout mutant, indicating that hyperpolarization increases colistin susceptibility in the *purA*-mutant. In addition, the cell surface charge, which plays an important role in colistin resistance, was not altered in the *purA* mutant. This study is the first to show that the disruption of *de novo* purine synthesis pathway increases colistin susceptibility via membrane hyperpolarization.

**Figure 6.**
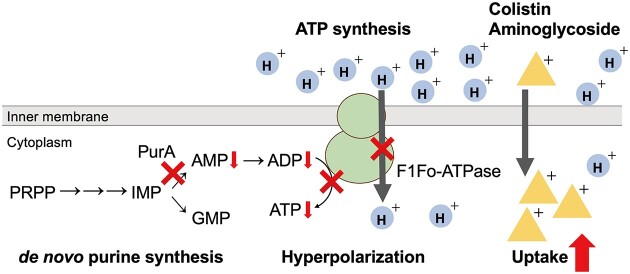
Model of increased colistin-susceptibility caused by *purA* knockout. *purA* knockout decreased AMP levels, which reduced the levels of ADP and ATP. ATP synthesis is coupled with proton transfer; therefore, decreased ATP synthesis causes membrane hyperpolarization. Membrane hyperpolarization increased the uptake of positively charged antimicrobials such as colistin and aminoglycosides, resulting in increased susceptibility to these molecules.

In the *purA*-knockout mutant, the amount of ATP was lower than that in the wild-type strain. Knockout of *purA* decreased AMP synthesis, resulting in the depletion of ADP and ATP. F1Fo-ATP synthase couples ATP synthesis and proton transfer; thus, decreased ATP synthesis decreases proton transfer, leading to hyperpolarization (Fig. 6). The membrane hyperpolarization could lead to increased uptake of colistin and aminoglycosides (Fig. 6). Accordingly, we identified the knockout mutants of *atpB, atpE*, and *atpH* that encode ATP synthase subunits, as colistin-sensitive mutants (Table S3). The knockout of ATP synthase subunits possibly decreases ATP synthesis, leading to the hyperpolarization of the membrane, resulting in colistin susceptibility. ATP synthase knockout leads to a colistin-sensitive phenotype in *S. aureus* (Vestergaard et al. 2017), indicating that colistin susceptibility in response to decreased ATP synthesis is conserved between gram-negative and gram-positive bacteria. However, as the purine synthesis pathway is closely related to various cellular processes, we cannot exclude the possibility that factors other than ATP synthesis or membrane hyperpolarization might be involved in colistin susceptibility. The effect of *purA*-knockout on the cellular processes other than the membrane potential should be investigated in future research.

For *Bacillus anthracis, Salmonella dublin*, and *Listeria monocytogenes, purA* knockout decreases bacterial cells in mouse tissues (Ivanovics et al. 1968, Sigwart et al. 1989, Faith et al. 2012). For *S. aureus, purA* knockout attenuates the bacterial killing activity in zebrafish (Connolly et al. 2017) and decreases bacterial cells in mouse tissues (Lan et al. 2010). For *E. coli, purA* knockout decreases bacterial growth in human serum (Samant et al. 2008). Thus, *purA* plays an important role in bacterial virulence. However, the mechanism by which *purA* contributes to virulence remains unclear. It is possible that the *purA*-knockout mutants have decreased ATP synthesis and are sensitive to the cationic antimicrobial peptides in human serum or animal tissues, resulting in decreased virulence. Further investigation is needed to examine the effect of the *purA*-knockout on the bacterial susceptibility to antimicrobial peptides during immunity.

The recent increase in colistin use has resulted in an increase in the number of colistin-resistant bacteria (Dadashi et al. 2022). This study suggests that *purA* knockout not only decreases bacterial virulence (Ivanovics et al. 1968, Sigwart et al. 1989, Faith et al. 2012, Connolly et al. 2017) but also sensitizes bacteria to colistin. Therefore, chemical molecules that target the *de novo* purine synthesis pathway could offer two benefits: decreased virulence and susceptibility to cationic antimicrobial peptides and aminoglycosides. In this regard, PurA is a promising antibiotic target.

## Supplementary Material

fnae007_Supplemental_File
