# Supplementary material for: Knockout of adenylosuccinate synthase purA increases susceptibility to colistin in Escherichia coli
Source: FEMS Microbiol Lett. 2024 Feb 1;371:fnae007. doi: 10.1093/femsle/fnae007 (PMC10876104; doi:10.1093/femsle/fnae007)
Supplement: fnae007_Supplemental_File [file fnae007_supplemental_file.docx]

| **Strain or Plasmid** | **Genotypes or characteristics** | **Source or reference** |
| --- | --- | --- |
| Strains |  |  |
| BW25113 | *rrnB,* Δ*lacZ*4787, *HsdR*514, Δ(*araBAD*)567, Δ(*rhaBAD*)568, *rph-1* | NBRP |
| JW4135-KC | BW25113 Δ*purA*::*kan* Kan^r^ | NBRP |
| JW3606-KC | BW25113Δ*waaG*::*kan* Kan^r^ | NBRP |
| TK0001 | BW25113 Δ*purA*::markerless | This study |
| JM109 | Host strain for cloning | Takara Bio |
| Plasmids |  |  |
| pMW118 | Low-copy-number plasmid; Amp^r^ | Nippon Gene |
| pMW118-purA | pMW118 with *purA*; Amp^r^ | This study |
| pCP20 | A temperature sensitive plasmid expressing FLP recombinase; Amp^r^ | EGSC |

**Table S1 Bacterial strains and plasmids used in this study.**

**Table S2 Primers used in this study.**

| purA_F_KpnI | GGTGGTACCATGACCAATTTGCCCGATAA |
| --- | --- |
| purA_R_BamHI | GGAGGATCCAGCGCACGTAATCCGTAATC |

**Table S3. List of genes of which deletion reduce colistin resistance**

| ID | Gene | Function |
| --- | --- | --- |
| JW3716-KC | *atpB* | ATP synthase subunit a |
| JW3715-KC | *atpE* | ATP synthase subunit c |
| JW3713-KC | *atpH* | ATP synthase subunit delta |
| JW3882-KC | *cpxA* | Sensor histidine kinase CpxA |
| JW1057-KC | *flgN* | Flagella synthesis protein FlgN |
| JW1224-KC | *galU* | UTP--glucose-1-phosphate uridylyltransferase |
| JW4216-KC | *holC* | DNA polymerase III subunit chi |
| JW2513-KC | *iscU* | Iron-sulfur cluster assembly scaffold protein IscU |
| JW2137-KC | *mglB* | D-galactose/methyl-galactoside binding periplasmic protein MglB |
| JW4135-KC | *purA* | Adenylosuccinate synthetase |
| JW3596-KC | *rfaC* | Lipopolysaccharide heptosyltransferase 1 |
| JW3595-KC | *rfaF* | ADP-heptose--LPS heptosyltransferase 2 |
| JW5413-KC | *rimM* | Ribosome maturation factor RimM |
| JW1644-KC | *rnt* | Ribonuclease T |
| JW3907-KC | *rpmE* | 50S ribosomal protein L31 |
| JW3261-KC | *rpmJ* | 50S ribosomal protein L36 |
| JW3563-KC | *selB* | Selenocysteine-specific elongation factor |
| JW5503-KC | *tolC* | Outer membrane protein TolC |
| JW5581-KC | *ubiE* | Ubiquinone/menaquinone biosynthesis C-methyltransferase UbiE |
| JW1559-KC | *ydfW* | Protein YdfW |
| JW2500-KC | *yfgA* | Cytoskeleton protein RodZ |
